# The Uses and Effects of Gude’s Manganiferous Iron Peptone (Liquor Mangano-Ferri Peptonatus Gude)

**Published:** 1892-12

**Authors:** 


### THE USES AND EFFECTS OF GUDE'S MANGANIFEROUS IRON PEPTONE (LIQUOR MANGANO-FERRI PEPTONATUS GUDE).

By DR. HEITZMANN.

[*Journal of Balneology and Dietary, September, 1892.*]

The employment of iron preparations, both in essential anemia (chlorosis) and in the symptomatic forms of this affection, produced by severe losses of blood, dates to the earliest times. Long before the chemical relation of this effect was known, these remedies were administered on the ground of pure empirical experience.

When Hannon pointed out the high significance of manganese, as well as of iron, with regard to the absorption of oxygen by the blood, and when this discovery was confirmed by Ruehle, efforts were made to produce, by combination of both remedies, preparations which would best fulfil the therapeutic indications in all directions.

Former attempts of this kind failed to give the desired results. The aim was to combine both metals in such a form as would enable them to be absorbed throughout the entire extent of the alimentary canal, and, at the same time, be devoid of disagreeable taste, which would prevent their prolonged administration. After a series of experiments made in this direction, I found, in the preparation discovered by Dr. A. Gude, the liquor mangano-ferri peptonatus, a remedy which fulfilled the above requisites, and can recommend it most heartily.

This liquor mangano-ferri peptonatus (Gude) is a clear, dark, wine-red fluid, having an agreeable, non-metallic, astringent taste. The latter property gives it a great advantage over other similar preparations, for the remedy is always taken with pleasure, and may, therefore, be administered for a long time without exciting the disgust of the patient. No irritation of the stomach is produced, nor is the digestion disturbed in the least respect; indeed, as regards the latter, a stimulation of the long absent appetite could be demonstrated within a short time.

The liquor mangano-ferri peptonatus (Gude), usually mixed with some water, is prescribed in doses of two or three dessertspoonfuls, increased to as many tablespoonfuls, per day. An especially agreeable manner of administration is by addition of cold milk, which then assumes a light chocolate color and an agreeable taste. Prescribed in this form, we obtain from this preparation everything that could be expected from a remedy for anemia. The tincture may also be mixed with white and sweet wines, excepting the red wines, which contain tannic acid, and an occasional change in the manner of administration is sometimes of advantage, especially in the case of children.

The diet, during the use of this preparation, should consist of milk, meats—especially ham—fowl, soft-boiled eggs, and other easily digested foods. On the other hand, sour and fatty foods, red wines, and raw fruits are to be avoided.

The remedy is to be administered for a number of weeks, especially in cases of chlorosis, but in the case of young girls, up to twelve years of age, it is best to commence with a daily dose of two teaspoonfuls (ten grammes). In adults, the dose of the tincture may be increased in a few days to one tablespoonful twice or thrice daily, or even to ten or twenty grammes. The preparation should be well protected from the light, and preserved in a cool place in a well-stoppered bottle.

I have employed the tincture with much success, both in chlorosis and in cases of anemia in girls and women, due to loss of blood, menorrhagia, metrorrhagia, inflammation of the pelvic organs, peri- and parametritis, or prolonged leucorrhea. In almost every instance I observed, within a short time, increase of appetite, improved nutrition, healthier color of the face, and increase of weight. I was surprised to learn how much more readily the liquor mangano-ferri peptonatus was taken than similar preparations, without ill effects, even after protracted use.

To illustrate my remarks, I will cite a few cases. I will first report a case of chlorosis, treated with this remedy, which was under constant observation :

The patient, a school girl, aged 16, began to menstruate one year ago, but, after appearing regularly for three periods, the flow suddenly ceased, probably in consequence of mental over-exertion, and symptoms of chlorosis soon developed. The various preparations of iron were tried, but were either not well borne or excited so much disgust that they were discontinued by the capricious patient. A milk cure was prescribed, but followed for only a short time. When, however, I resorted to the liquor mangano-ferri peptonatus (Gude) I was surprised to find that the girl took it willingly, and that it was well borne. She made a rapid recovery, and, after the use of two bottles, had regained her former healthy color, while her strength and menstruation returned.

Case II. A married lady, aged 24, had acquired—apparently of abortion at a very early period—an immense peri- and parametritis with an exudation of the size of a child's head. The latter disappeared almost completely under suitable treatment and rest, so that only a slight induration was present in the parametrium after three weeks. Owing to the considerable anemia and loss of appetite, however, the patient recovered very slowly, and, for this reason, I ordered the liquor mangano-ferri peptonatus (Gude). A few days after its use the appetite reappeared, recovery ensued rapidly, and five weeks later her health was completely restored.

Case III. A married lady, aged 30, had suffered from leucorrhea, due to catarrhal inflammation of the vagina for two years, and, although the local trouble had been much relieved, she continued pale and weak. As her chlorotic daughter, at the time, was taking the liquor mangano-ferri peptonatus (Gude) with marked benefit, I advised her also to try this preparation. She followed my advice, and after fourteen days the weak, sluggish, and pale woman seemed as if transformed. She has since regained her former health.

These few cases, which were under continued observation, will confirm what has been said above regarding the manner of application and the effect of the liquor mangano-ferri peptonatus. I regard it as superfluous to cite other cases, since a few closely observed cases teach more than a host of superficial observations.

On the ground of my experience, I consider myself warranted in directing the attention of physicians to this remedy, and feel convinced that further trials will give equally favorable results. Even in cases where local treatment is necessary, the liquor mangano-ferri peptonatus (Gude) will prove a valuable auxiliary in our treatment.—*Allgemeine Wiener medizinische Zeitung*, xxxvi.

